# Dataset on the single nucleotide variation in diversity panel of 500 lettuce accessions genotyped with tunable genotyping-by-sequencing (tGBS) method

**DOI:** 10.1016/j.dib.2023.109419

**Published:** 2023-07-17

**Authors:** Ivan Simko

**Affiliations:** U.S. Department of Agriculture, Agricultural Research Service, Crop Improvement and Protection Research Unit, Salinas, CA 93905, United States

**Keywords:** *Lactuca*, Molecular markers, Genome-wide association study, Marker-assisted selection

## Abstract

Lettuce (*Lactuca sativa* L.) is an important leafy vegetable cultivated in moderate climates around the world. Based on phenotypic characteristics, such as formation and size of ‘heads’ (leaves arranged in a dense rosette), size, and texture of leaves, length of stems, and size and composition of seeds, lettuce cultivars can be classified into several distinct horticultural types (Batavia, butterhead, iceberg, Latin, leaf, oilseed, romaine, and stem). *L. serriola*, a wild progenitor of cultivated lettuce, is frequently utilized in breeding programs to introgress desirable genes and alleles (e.g., resistance to diseases) into lettuce gene pool. A diversity panel of ∼500 lettuce accessions was genotyped with tunable genotyping-by-sequencing (tGBS) method to identify single nucleotide polymorphism (SNP) sites. 115,261 SNPs were positioned on lettuce genome using the reference genome of cultivar Salinas. The described diversity panel together with the set of SNP markers can be used for mapping quantitative trait loci (QTL) and to develop marker assays for marker assisted selection (MAS). Identified SNP sites can also be used to identify F_1_ hybrids, genotype gene bank collections, and in other areas of lettuce genetics and breeding.

**Specifications Table**

**Table:** 

Subject	Horticulture
Specific subject area	Genotyping-by-sequencing
Type of data	Tables, sequence data for 115,261 aligned SNPs generated for 514 lettuce samples from 498 lettuce accessions.
How the data were acquired	Seeds from 498 lettuce accessions were planted into ∼15 cm in diameter pots and grown in a greenhouse until plants matured and produced seeds. Twenty-five of the harvested seeds per accession were sent to Data2Bio (Ames, Iowa, USA) for DNA extraction and sequencing using tunable genotyping-by-sequencing (tGBS) technology [1].
Data format	Raw Filtered
Description of data collection	Sequencing was performed on Ion Proton System (ThermoFisher Scientific, Waltham, Massachusetts, USA). Sequences were aligned to the lettuce reference genome of cv Salinas, version 8 [2].
Data source location	Seeds of all accessions were obtained from the seedbank of USDA-ARS in Salinas, California, USA.
Data accessibility	Repository name: European Nucleotide Archive (ENA) at the European Molecular Biology Laboratory of the European Bioinformatics Institute (EMBL-EBI) Data identification number: PRJEB63154 Direct URL to data: https://www.ebi.ac.uk/ena/browser/view/PRJEB63154
Related research article	I. Simko, H. Peng, J. Sthapit Kandel, R. Zhao R, Genome-wide association mapping reveals genomic regions frequently associated with lettuce field resistance to downy mildew, Theor. Appl. Genet. 135 (2022) 2009–2024. https://doi.org/10.1007/s00122–022–04090–3

## Value of the Data

• The tGBS markers can be used in linkage and association mapping studies, analyses of population structure, and developing assays for marker-assisted selection (MAS).
• These data will benefit researchers studying inheritance and genetics of complex traits, lettuce breeders utilizing marker-assisted breeding, and gene bank curators analyzing inventories.
• The tGBS markers can further be used to identify F_1_ hybrids, construct haplotypes, capture diversity in lettuce horticultural types, predict the breeding value in genomic selection, and determine genetic differences within and among populations.
• SNP data from two *L. indica* accesions can provide an introductory insight into the genetic similarity with *L. sativa* and *L. serriola*.

## 1 Objective

The main objective for generating tGBS markers using the diversity panel of ∼500 lettuce accessions was to apply genome-wide association studies (GWAS) to map quantitative trait loci (QTL) for resistance to Impatiens necrotic spot virus (INSV) [3], downy mildew [4] bacterial leaf spot [5], lettuce drop, yellow spot malady [6], plant development, postharvest quality [7], content of anthocyanins and chlorophylls, and other economically important traits. Seeds of genotyped accessions are available for distribution to collaborators.

## 2 Data Description

SNP data were generated for 514 samples from 498 lettuce accessions. Analysis of sequencing data identified 2,314,942 polymorphic sites. The number of SNPs was subsequently filtered using the minimum calling rate (MCR) cut-off. MCR_20_ that keeps only SNPs which were genotyped in at least 20% of all sequenced samples (≥ 103 samples), maintained 115,261 SNPs [8]. Further increase in the cut-off value gradually decreased the number of SNPs to 182, which were detected when sequencing data from all 514 samples (MCR_100_) were available for analysis (Table 1).

**Table 1:** Effect of the minimum calling rate (MCR) on the number of remaining SNPs.

Minimum calling rate	Number of remaining SNPs
Total polymorphic sites^a^	2,314,942
MCR_20_	115,261
MCR_50_	50,325
MCR_60_	39,762
MCR_70_	30,114
MCR_80_	21,398
MCR_90_	12,549
MCR_100_	182

a No limit.

**Fig. 1:**
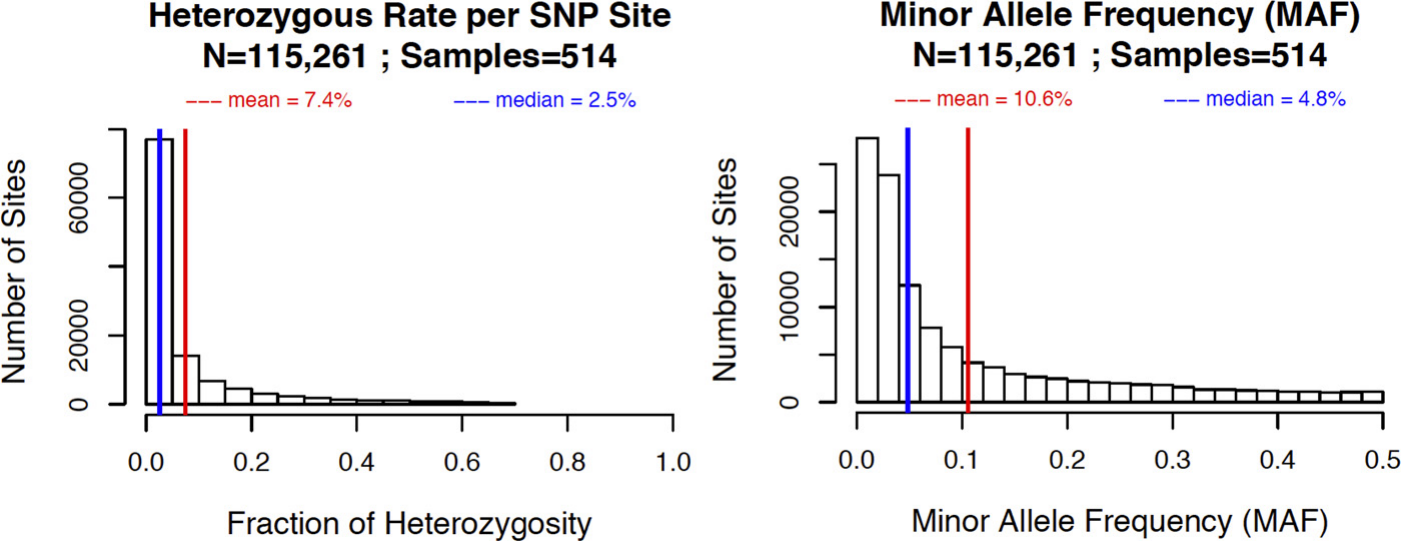
Heterozygous rate per SNP site and minor allele frequency (MAF) in 514 lettuce samples as determined by 115,261 tGBS markers. Red lines and related numbers show mean values, while blue lines and related numbers indicate median values for the set.

**Fig. 2:**
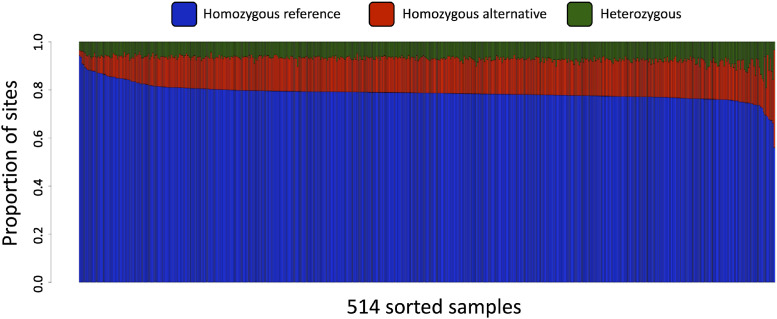
Proportion of genotypes in each of 514 lettuce samples as determined by 115,261 tGBS markers. Missing data were not considered.

**Fig. 3:**
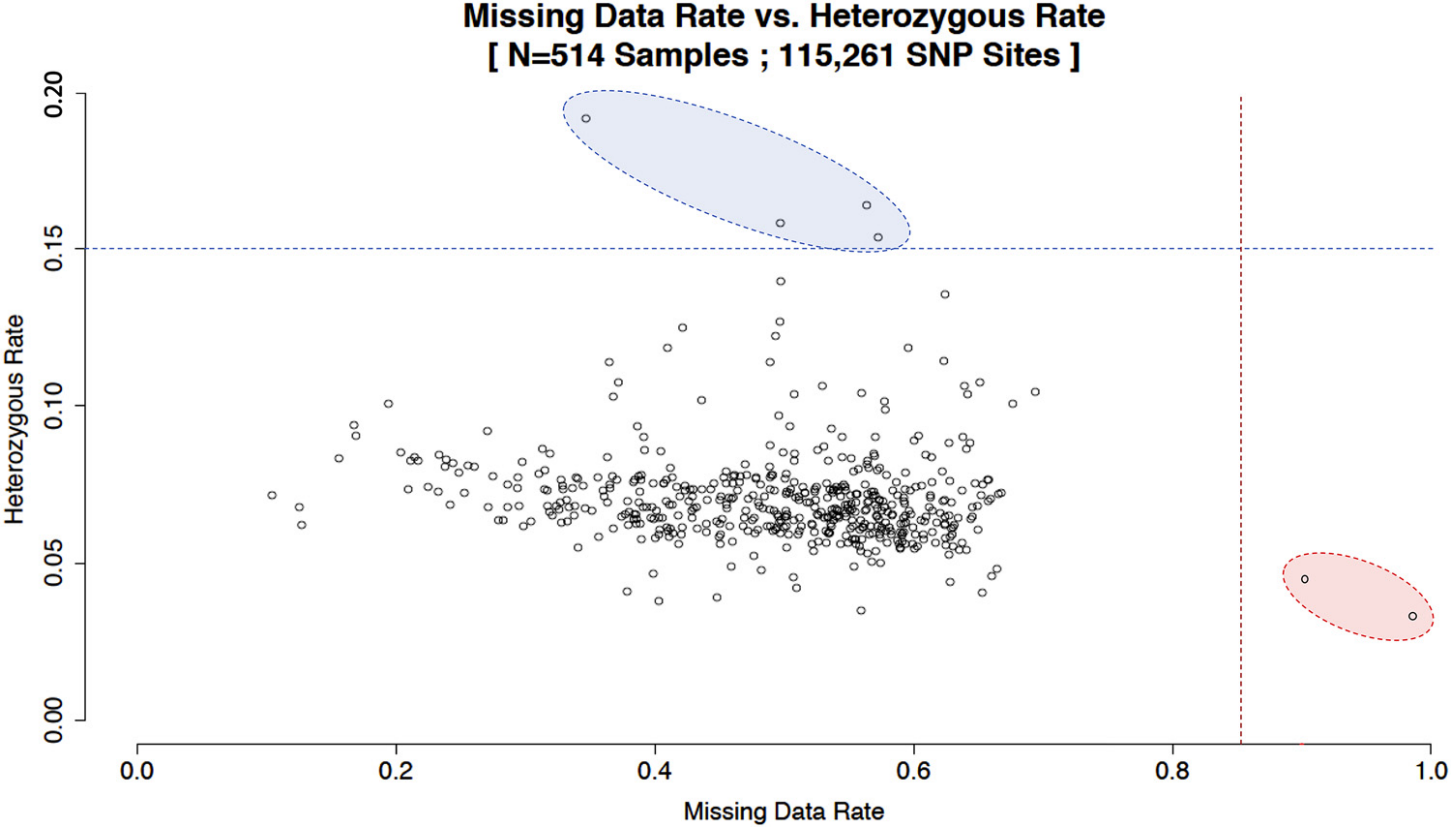
Missing data rate versus heterozygous rate in 514 lettuce samples as determined by 115,261 tGBS markers. The dashed, horizontal blue line indicates heterozygous rate of 15%, the blue oval highlights four samples with the highest heterozygous rate. The dashed, vertical red line indicates missing data rate of 85%, the red oval highlights two samples with the highest missing data rate.

Analysis of 115,261 SNPs identified in 514 samples using MCR_20_ determined that the mean heterozygous rate per SNP site was 7.4% and the mean minor allele frequency (MAF) was 10.6% (Fig. 1). Proportion of genotypes homozygous for an allele present in the reference genome showed a normal-like distribution (Fig. 2). As expected, two *L. indica* samples (GBS-329A and GBS-330A) had the highest rate of missing data (> 85%). Four samples (GBS-430A, GBS-440A, GBS-459A, and GBS507A) showed heterozygous rate > 15%, indicating that they are genetically substantially less uniform than other tested samples (Fig. 3).

When this diversity panel is used for GWAS, it is recommended to combine tGBS data from samples that were replicated (four GBS-373A samples, two GBS-124A samples, and two GBS-267A samples) or use only one of the replicated samples as a representative for the respective genotype. In addition, it is recommended to eliminate 11 samples that combine seeds from two phenotypically distinct lines (GBS-385A, GBS-394A, GBS-397A, GBS-437A, GBS-440A, GBS-452A, GBS-478A, GBS-481A, GBS-483A, GBS-505A, and GBS-507A). For example, the combined sample GBS-385A should be eliminated, but two individual, distinct phenotypes GBS-385A_B (black seeds) and GBS-385A_W (white seeds) should be kept. After eliminating data from two *L. indica* accessions (GBS-329A and GBS-330A), the diversity panel will contain data for 496 unique accessions. Depending on a project objective, data for four *L. serriola* accessions (GBS-459A, GBS-533A, GBS-547A, and GBS-548A) could be kept or eliminated.

## 3 Experimental Design, Materials and Methods

Cultivated lettuce (*Lactuca sativa* L.) is a diploid (*2n* = *2x* = 18) autogamous species with highly homozygous and homogenous commercial cultivars. A lettuce diversity panel consisting of 498 accessions was developed that includes commercial cultivars, advanced breeding lines, plant introductions, and selected individuals from recombinant inbred lines (RIL). The panel comprised 492 accessions from eight lettuce horticultural types (Batavia, butterhead, iceberg, Latin, leaf, oilseed, romaine, and stem) though most of the accessions phenotypically resembled romaine type. In addition, four accessions of *L. serriola*, a close relative of cultivated lettuce used in breeding programs, were included in the diversity panel. Two accessions of *L. indica* were also genotyped but should not be used in GWAS due to their sexual incompatibility with cultivated lettuce.

Seeds of each accession were planted into ∼15 cm in diameter pots and grown in a greenhouse. The next generation of seeds was harvested from only a single mature plant per accession to minimize a possibility of seed mix-up. Twenty-five of the harvested seeds from each accession were used for DNA extraction and genotyping. Additional 16 samples were added to the diversity panel as internal checks (replicates of individual accessions and combined seed lots), therefore a total of 514 seed samples were sent for DNA extraction and sequencing to Data2Bio (Ames, Iowa, USA).

Genotyping was performed using tGBS technology that mitigats the high rate of genotypic errors and missing data that occurs at the conventional GBS approach [1]. In the tGBS method of genome reduction, two restriction enzymes are used to generate overhangs in opposite orientations. Single-strand oligos, instead of double-stranded adapters used in conventional GBS [9], are ligated to these overhangs, thus ensuring that only double digested fragments are amplified and sequenced [1]. Consequently, sequencing reads from tGBS libraries that are highly enriched at target sites produce a higher average read depth (and accuracy) per target site than conventional GBS, at the same number of reads per sample [1]. Three sequencing runs were performed on an Ion Proton System (ThermoFisher Scientific, Waltham, Massachusetts, USA) to generate raw data reads. Prior to alignment, the nucleotides of each raw read were scanned for Phred quality [10,11]. Sequence reads with the Phred quality scores less than 15 (error rate > 3%) were trimmed using LUCY2 [12], thus eliminating 11.1% of base pairs. The trimmed reads from each sample were aligned to the nine chromosomes of the reference genome of cultivar Salinas v.8 [2] using GSNAP [13].

Confidently mapped reads were filtered and used for SNP discovery. SNP calling was conducted using the uniquely aligned reads after examining 3,438,949 bases that had ≥ 5 reads in at least 50% of the samples (257 individual samples). Polymorphism were ignored if detected in the first 3 bp and the last 3 bp of each read. SNP was determined to be homozygous if the most common allele was supported by at least 80% of all the aligned reads covering that position, at least five unique reads supported the most common allele, and each polymorphic base had the Phred quality score ≥ 20 (error rate ≤ 1%). SNP was determined to be heterozygous if the two most common alleles were supported by at least 30% of all aligned reads covering that position, at least five unique reads supported each of the two most-common alleles, sum of reads of the two most common alleles accounted for at least 80% of all aligned reads covering that nucleotide position, and each polymorphic base had the Phred quality score of ≥ 20 (error rate ≤ 1%).

## Ethics Statements

The current work meets the ethical requirements for publication in Data in Brief. The current work does not involve human subjects, animal experiments, or any data collected from social media platforms.

## CRediT authorship contribution statement

**Ivan Simko:** Conceptualization, Methodology, Software, Validation, Formal analysis, Investigation, Resources, Data curation, Writing – original draft, Writing – review & editing, Visualization, Supervision, Project administration, Funding acquisition.

## Declaration of Competing Interest

The author declares no known competing financial interests or personal relationships that could have appeared to influence the work reported in this paper.

## Data Availability

PRJEB63154, Lettuce Genetic Diversity (Original data) (European Nucleotide Archive (ENA)).
